# The impact of age and sex on the inflammatory response during bone fracture healing

**DOI:** 10.1093/jbmrpl/ziae023

**Published:** 2024-02-22

**Authors:** Kristin Happ Molitoris, Abhinav Reddy Balu, Mingjian Huang, Gurpreet Singh Baht

**Affiliations:** Department of Orthopaedic Surgery, Duke Molecular Physiology Institute, Department of Pathology, Duke University, Durham, NC 27701, United States; Feinberg School of Medicine, Northwestern University, Chicago, IL 60611, United States; Department of Orthopaedic Surgery, Duke Molecular Physiology Institute, Department of Pathology, Duke University, Durham, NC 27701, United States; Department of Orthopaedic Surgery, Duke Molecular Physiology Institute, Department of Pathology, Duke University, Durham, NC 27701, United States

**Keywords:** aging, cytokines, fracture healing, immune cells, inflammation, sex

## Abstract

Inflammation is thought to be dysregulated with age leading to impaired bone fracture healing. However, broad analyses of inflammatory processes during homeostatic bone aging and during repair are lacking. Here, we assessed changes in inflammatory cell and cytokine profiles in circulation and in bone tissue to identify age- and sex-dependent differences during homeostasis and repair. During homeostatic aging, male mice demonstrated accumulation of CD4+ helper T cells and CD8+ cytotoxic T cells within bone while both pro-inflammatory “M1” and anti-inflammatory “M2” macrophage numbers decreased. Female mice saw no age-associated changes in immune-cell population in homeostatic bone. Concentrations of IL-1β, IL-9, IFNγ, and CCL3/MIP-1α increased with age in both male and female mice, whereas concentrations of IL-2, TNFα, TNFR1, IL-4, and IL-10 increased only in female mice – thus we termed these “age-accumulated” cytokines. There were no notable changes in immune cell populations nor cytokines within circulation during aging. Sex-dependent analysis demonstrated slight changes in immune cell and cytokine levels within bone and circulation, which were lost upon fracture injury. Fracture in young male mice caused a sharp decrease in number of M1 macrophages; however, this was not seen in aged male mice nor in female mice of any age. Injury itself induced a decrease in the number of CD8+ T cells within the local tissue of aged male and of female mice but not of young mice. Cytokine analysis of fractured mice revealed that age-accumulated cytokines quickly dissipated after fracture injury, and did not re-accumulate in newly regenerated tissue. Conversely, CXCL1/KC-GRO, CXCL2/MIP-2, IL-6, and CCL2/MCP-1 acted as “fracture response” cytokines: increasing sharply after fracture, eventually returning to baseline. Collectively, we classify measured cytokines into three groups: (1) age-accumulated cytokines, (2) female-specific age-accumulated cytokines, and (3) fracture response cytokines. These inflammatory molecules represent potential points of intervention to improve fracture healing outcome.

## Graphical Abstract

**Graphical Abstract:**
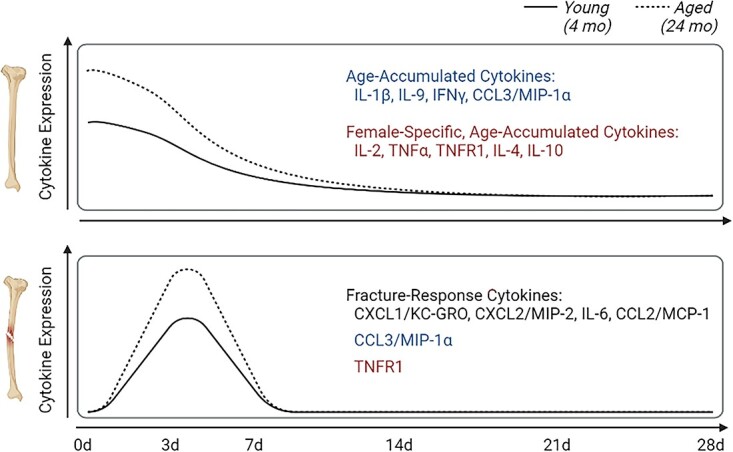

## Introduction

Advanced age is associated with elevated, chronic inflammation during homeostasis and with dysregulated inflammation in response to injury.^1–6^ Bone fracture injury triggers both local and systemic inflammation leading to activation of hematopoietic cells and production of cytokines, which induce mesenchymal progenitor cell recruitment and formation of the cartilaginous and eventually of the bony callus.^7–10^

Age-associated dysregulation of the initial inflammatory phase of bone fracture repair leads to improper cellular recruitment and signaling, dysfunction of progenitor cell differentiation and activity, and subsequently diminished fracture healing.^11–14^ Similar immune-regulated mechanisms have been implicated in decreased bone health and regeneration seen in female vs male patients.^15^^,^^16^

T cells have been shown to be critical in normal fracture repair^17–19^; work by us and others has demonstrated that age-associated dysregulation in macrophage recruitment and in differentiation during fracture healing leads to an increase in cytokine production at the injury site.^20–23^ Therefore, this first stage of healing presents a potential time for therapeutic intervention to improve aged bone healing. Indeed, when we used a small-molecule resolving agent to reverse these age effects, macrophage phenotype was rejuvenated, cytokine production was decreased, and fracture healing was improved.^24^

While such immunomodulatory interventions have the potential to improve tissue regeneration, broad investigations identifying specific age- or sex-dependent changes in inflammatory processes are lacking. Here, we used young and aged mouse models of tibial fracture injury to identify the age- and sex-associated changes in immune cell numbers and in cytokine levels within the healing fracture callus and in circulation. This investigation was carried out longitudinally allowing analysis prior to fracture injury and during all stages of bone repair. We identified age- and sex-dependent alterations in inflammatory cell population and in cytokine abundance secondary to injury.

## Materials and methods

### Mouse models

All procedures were approved by the Duke Institutional and Animal Care and Use Committee. Mice were purchased from Jackson Labs (C57BL/6 J—stock No. 000664) and aged 4 months (“young” mice) or 24–26 months (“old” mice).

### Tibial fracture surgery

Fractures were performed as previously described.^25–27^ Briefly, mice were anesthetized using an induction chamber with 2% isoflurane and an oxygen flow rate of 1 L/minute. Sedation was ensured using paw-pinch test and the animal was placed on the surgical area. A nose cone was used to maintain sedation of the mouse using a flow rate of 500 mL/minute oxygen with 2% isoflurane. Throughout the procedure sedation was ensured using paw-pinch test. The surgical area proximal to the knee was shaved and disinfected. Following an incision, a hole was drilled into the tibial plateau and a 0.7 mm stainless steel pin was placed into the medullary cavity and cut flush with the tibial plateau. A tibial fracture was induced mid-shaft using blunt scissors and the incision was closed using wound clips. For analgesia, 0.5 mg/kg buprenorphine-sustained release was administered subcutaneously at the beginning of the procedure. Fracture calluses were harvested either 3, 7, 14, 21, or 28 days post-fracture (dpf). Additionally, from non-surgical mice, intact tibiae were collected for all groups.

### Analysis of fracture healing

Fracture healing was assessed at 21dpf by μCT or at 28dpf by mechanical testing. For μCT analysis, fracture calluses were dissected and fixed in 10% Zn-formalin at room temperature for 5 days. μCT analysis was conducted using a Scanco vivaCT 80 (Scanco Medical, Brüttisellen, Switzerland) at a scan resolution of 8 μm. Calluses were scanned 1 mm proximal and 1 mm distal to the fracture site and assessed for total volume (TV) and bone volume (BV) in mm^3^, and ratio of bone volume to total volume (BV/TV).^28^ For mechanical testing, fracture calluses were wrapped in PBS-soaked gauze and stored at –80°C. In preparation for mechanical testing, tibiae were brought to room temperature and tested in four-point bending in the medial–lateral direction with the medial side in tension using an ElectroForce 3220 Series III instrument (TA Instruments, New Castle, USA). Cylindrical rollers 2 mm in diameter were used to apply four points of loading. The midpoints of the top two rollers were positioned 4 mm apart while the midpoints of the bottom two rollers were positioned 10 mm apart. The top fixture was able to tilt horizontally to allow for simultaneous contact between all four rollers and the sample. To position each tibia within the four-point bending fixture, the most proximal location of the tibia–fibula junction was aligned with the outer edge of one of the bottom-loading rollers, ensuring the fracture callus was centered among all rollers. Bending failure tests were performed in displacement control at a rate of 0.025 mm/second. Testing was terminated by failure, as determined by a 95% drop in load.

Load, displacement, and time were recorded at a sampling frequency of 10 Hz. Structural stiffness (N/mm) was calculated as the slope of the load versus displacement data between 30% and 70% of the load at failure, to exclude nonlinear toe-regions in the force–displacement curves. Force to fracture was identified as the maximal load that occurred prior to failure.

### Flow cytometry

**Figure 1:**
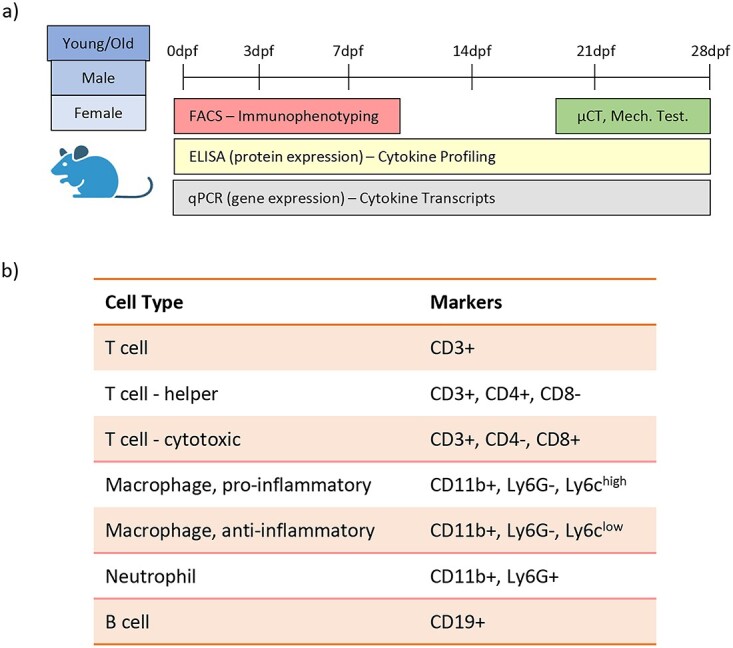
Study design. (A) Young and old male and female mice underwent tibial fracture surgery and immunophenotyping was carried out for local fracture callus tissue and for systemic blood. Immunophenotyping involved analysis of inflammatory cells, inflammatory cytokines, and cytokine transcripts. (B) Cell surface markers were used to define inflammatory cell sub-types.

Midshafts of unbroken tibiae (0dpf), fracture calluses (3 and 7dpf), and blood from each animal were analyzed for flow cytometry as described previously.^24^ Briefly, unbroken bone (0dpf) and calluses (3 and 7dpf) were manually dissected into 1-mm^2^ pieces, washed with phosphate buffered saline (PBS), and digested with Collagenase type I (0.2 mg/mL, Gibco #1710017) for 90 minutes at 37°C. Following digestion, debris was removed using a 70 μm cell strainer. Cells were pelleted and resuspended in PBS with 1% FBS (PBS+) for staining. Blood samples were collected in ethylenediaminetetraacetic acid (EDTA)-vacuette tubes (Grenier Bio-One, Monroe, North Carolina) and centrifuged at 350 g for 10 minutes at 4°C. The plasma was collected and washed twice with 10 ml of Red Blood Cell Lysis Buffer (Biolegend, San Diego, California). Pellets were resuspended in PBS+ for staining. Fluorescence-conjugated antibodies [CD11b (#101216), Ly6C (#128016), Ly6G (#127626), CD19 (#115508), CD3 (#100210), CD4 (#100434), and CD8b (#140422); 1:500; Biolegend] were added and incubated at 4°C for 30 minutes. Cells were then washed twice using PBS+ and assessed using an Attune Nxt Flow Cytometer (Thermo Fisher, Waltham, USA). Total “helper” and “cytotoxic” T cells, total pro-inflammatory (referred to as “M1”) and anti-inflammatory (referred to as “M2”) macrophages, neutrophils, and B cells were identified with primary fluorescent conjugated antibodies as shown in Figure 1. Stained cells were counted and expressed as a percentage of total live single cells. Unstained cells were used to determine background.

### Immunoassay/cytokine analysis

Inflammatory and angiogenic biomarkers and cytokines were quantified by enzyme-linked immunosorbent assay (ELISA). Fracture calluses were pulverized in liquid nitrogen and radio immunoprecipitation assay (RIPA) lysis buffer (Millipore Sigma, Burlington, Massachusetts, USA) was used to extract proteins as previously described.^26^^,^^29^ Blood was harvested into EDTA-vacuette tubes, centrifuged for 10 minutes at 350 *g* at 4°C, and plasma collected for analysis.

Custom multiplex assays were performed to measure mouse cytokines (IL-1β, IL-9, IFNγ, CCL3/MIP1α, IL-2, TNFα, TNFR1, IL-4, IL-10, CXCL1/KC-GRO, CXCL2/MIP-2, IL-6, CCL2/MCP-1) (MesoScale Discovery, Gaithersburg, USA) in plasma (diluted 2-fold) and fracture callus samples. Mean reported intra- and inter-assay coefficients of variation for these assays are all <7% and <12%, respectively. For samples having values below the lower limit of detection (LLOD), one half of LLOD was imputed for these values for the purposes of statistical analyses. Enzyme-linked immunosorbent assay procedures were carried out by the Duke Biomarkers Core.

### Reverse transcription polymerase chain reaction

Tissue samples were harvested, immersed immediately into liquid nitrogen, and pulverized into powder prior to RNA extraction. Total RNA was isolated using TRIzol Reagent (Invitrogen Inc., Waltham, Massachusetts, USA) then purified with RNeasy Mini Kit (Qiagen, Germantown, Maryland, USA) and purity and quantity of RNA was determined using spectrometric methods. cDNA template was generated using random hexamers. Reverse transcription polymerase chain reaction (RT-PCR) was performed using PowerUp SYBr Green Master Mix (Applied Biosystem, Foster City, CA, USA) and primers for *Ifng, Il1b, Il9* (sets 1–3), *Ccl3/Mip1a, Cxcl1/Kcgro, Cxcl2/Mip2, Il6, Ccl2/Mcp1* were purchased from Integrated DNA Technologies (Coralville, Iowa, USA). Transcript levels in samples were investigated using a QuantStudio 6 Real-Time PCR System and compared using the ΔΔCt method to the transcript of glyceraldehyde-3-phosphate dehydrogenase (*Gapdh*) as a housekeeping control. All sequences are listed in Supplementary Table S1. A minimum of four replicates of all samples were analyzed.

### Statistical analysis

All data are expressed as mean ± standard deviation. Flow cytometry and quantitative real-time PCR data were analyzed with two-tailed t-test for singular comparisons. Two-way analysis of variance (ANOVA) was performed for ELISA data using GraphPad Prism 9 followed by Tukey’s (post-hoc) test for multiple comparisons. Confidence level for statistical significance is as noted within the figure or figure legend. For all figures, blue represents young males (YM); brown represents old males (OM); purple represents young females (YF); red represents old females (OF).

## Results

### Fracture healing is diminished by advanced age

We performed tibial fracture surgery on young (4-month-old) and old (24–26-month-old) male and female mice and assessed bone healing using μCT analysis and mechanical testing. μCT analysis at 21dpf determined total volume (TV), bone volume (BV), and calculated ratio of bone volume (BV) of the fracture callus. As we had previously shown, bone-healing metrics diminished with age^26^^,^^30^; however, sex played a major role in fracture-healing metrics. The TV of the fracture calluses did not differ by age in neither males nor females; however, males did display consistently larger fracture calluses (11.5 mm^2^ for males and 9.2 mm^2^ for females – Supplementary Figure S1A). Both BV and BV/TV decreased by approximately 20% with age in both males and females (Supplementary Figure S1B and C). Mechanical testing assessed the quality of bone healing by determining the structural stiffness and force to refracture of 28-day-old healing tibiae. With advanced aged, structural stiffness decreased by approximately 20% in males and by 15% in females (Supplementary Figure S1D). Similarly, aged mice displayed approximately a 25% decrease in force to refracture in males and a 10% decrease in females (Supplementary Figure S1E). Importantly, while fracture calluses from both young and old males contained less relative bone volume than their female counterparts (Supplementary Figure S2C), fracture calluses from male mice demonstrated higher structural stiffness and force to refracture (Supplementary Figure S2D and E). Collectively, these findings demonstrate that bone fracture healing is impaired by advanced age.

### Advanced age coincides with changes in immune cell populations in bone

Elevated, chronic inflammation is seen with aging and associated with age-associated shortcomings in tissue regeneration. We collected tibiae and blood from uninjured young and aged, male and female mice to establish the local and systemic inflammatory cell population changes in inflammaging in bone (Figure 1A). We determined the relative amounts of inflammatory cells in bone tissue and circulation using flow cytometry (Figure 1B).

**Figure 2:**
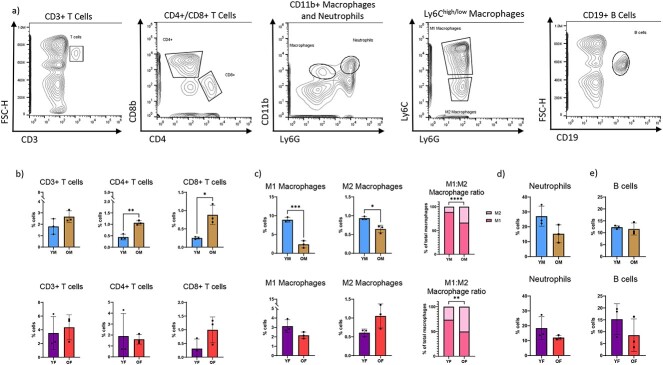
Inflammatory cells within bone are dysregulated with age. Tibial diaphyses from uninjured mice were homogenized and investigated for inflammatory cell populations using flow cytometry and related to total number of cells present within tissue. (A) Representative gating strategies shown. Total T cell number was measured using CD3 as a pan T cell marker; CD4+ helper and CD8+ cytotoxic T cell numbers were assessed. Macrophages were identified as CD11b+, Ly6G-. Pro-inflammatory macrophages (M1; Ly6c^high^) and anti-inflammatory macrophages (M2; Ly6c^low^) were assessed. Neutrophils were quantified as CD11b+, Ly6G+ cells and B cells were identified as CD19+ cells. (B) Percentage of CD3+, CD4+, and CD8+ T cells in the intact tibial diaphyses. (C) M1 and M2 macrophages were measured and ratio of M1:M2 was determined. (D) Neutrophils and (E) B cells were measured within intact tibial diaphyses. For all, *n* = 3; two-tailed t-tests were conducted. ^*^P < 0.05, ^*^^*^P < 0.01, ^*^^*^^*^P < 0.001, ^*^^*^^*^^*^P < 0.0001 (YM, young males; OM, old males; YF, young females; OF, old females).

The midshafts of unfractured tibiae were dissected and digested to generate single-cell suspensions. While there was no change in the number of total CD3+ T cells with advanced age, the number of CD4+ helper T cells and CD8+ cytotoxic T cells more than doubled with age in male mice but was not significantly different in female mice (Figure 2B). Macrophage phenotype was also affected by advanced age – there was an age-dependent decrease in the number of both pro-inflammatory “M1” macrophages (CD11b+, Ly6G-, Ly6c^high^) and anti-inflammatory “M2” macrophages (CD11b+, Ly6G-, Ly6c^low^) in male mice but not female mice (Figure 2C).^31^ Importantly, in both male and female mice, the M1:M2 ratio shifted toward M2 macrophages with advanced age (Figure 2C). Although neutrophil populations (Cd11b+, Ly6G+) decreased slightly in number with age in both male and female mice, there was no statistical difference between young and old bones (Figure 2D). Likewise, B cells (CD19+) did not display any significant age-dependent changes in bone (Figure 2E). Sex-dependent analysis of homeostatic bone revealed differences only in macrophage populations between male and female mice. The bones of young females contained lower amounts of M1 and M1 macrophages, however, this difference was lost with age (Supplementary Figure S3).

Similar to bone-tissue analysis, we immunophenotyped isolated peripheral blood mononuclear cells (PBMCs) from blood collections of young and aged mice. The total number of CD3+ T cells and CD4+ helper T cells in circulation decreased significantly in female mice but remained unchanged with advanced age in male mice (Supplementary Figure S4A). Conversely, with advanced age the number of CD8+ cytotoxic T cells increased significantly in male mice but remained unchanged in female mice (Supplementary Figure S4A). The number of circulating M1 macrophages decreased with advanced age in male mice but not in female mice while the number of M2 macrophages did not change significantly with age in male nor in female mice (Supplementary Figure S4B). Neutrophils exhibited an age-dependent decrease in male but not in female mice (Supplementary Figure S4C). B cells in circulation increased with age in male mice but decreased with age in female mice (Supplementary Figure S4D).

This PBMC analysis was also performed using sex as a variable. Young female circulation contained higher amounts of CD3+ and CD4+ helper T cells relative to the circulation of young males (Supplementary Figure S5A). Conversely, M1 macrophages and neutrophils were lower in the circulation of young females versus young males (Supplementary Figure S5B and C). Importantly, all of these differences were lost with age. The only sex-dependent difference in aged mice was that in B cells: the circulation of aged females contained lower amounts of B cells than did the circulation of aged males (Supplementary Figure S5D).

Collectively, these data confirm that there is an age-associated modulation of inflammatory cells within homeostatic bone tissue and in circulation. Furthermore, these age-associated changes vary depending on the sex of the animal.

### Age-associated changes in inflammatory-cell response during fracture injury

We performed tibial fracture surgery on young and aged mice to investigate age-associated changes in the inflammatory response to injury. Flow cytometry was performed on samples from calluses and blood during the initial stages of fracture healing (0, 3, and 7dpf – Figure 1A).

**Figure 3:**
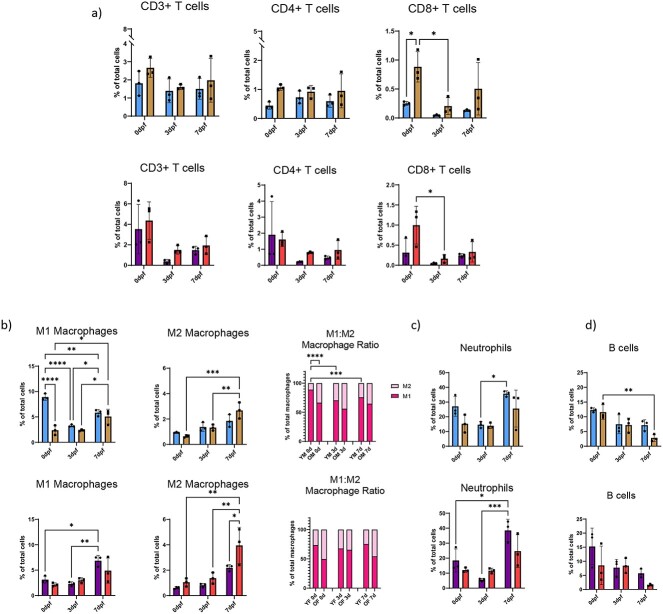
Advanced age alters inflammatory response to fracture injury. Mice underwent tibial fracture surgery and fracture calluses were harvested 0dpf (prior to fracture), 3dpf, and 7dpf. Calluses were homogenized and investigated for inflammatory cell populations using flow cytometry and related to total number of cells present within the fracture callus. (A) Total T cell number was measured using CD3 as a pan T cell marker; CD4+ cytotoxic and CD8+ helper T cell numbers were assessed within the CD3+ T cell population. (B) Pro-inflammatory macrophage (CD11b+, Ly6G-, Ly6c^high^) and anti-inflammatory macrophage (CD11b+, Ly6G-, Ly6c^low^) number was measured and M1:M2 ratio was determined. (C) CD11b+, Ly6G+ neutrophils, and (D) CD19+ B cells amounts were measured within fracture calluses. *N* = 3; data analyzed by two-way ANOVA followed by Tukey’s test when significant; ^*^P < 0.05, ^*^^*^P < 0.01, ^*^^*^^*^P < 0.001, ^*^^*^^*^^*^P < 0.0001 (YM, young males; OM, old males; YF, young females; OF, old females).

Surprisingly, no significant changes were seen in the number of CD3+ T cells nor of CD4+ helper T cells neither in the response to bone injury nor during the repair process (Figure 3A). In young mice, fracture did not change the number of CD8+ cytotoxic T cells; however, in aged mice, CD8+ cytotoxic T cell number decreased immediately after injury and remained decreased through to 7dpf (Figure 3A).

The number of M1 macrophages within the fracture callus decreased immediately after fracture injury in young, male mice while in female mice and in aged mice this number was unchanged immediately after injury (Figure 3B). The number of fracture callus M2 macrophages remained unchanged after injury in young mice; however, the M2 macrophage proportion significantly increased by 7dpf in all aged mice (Figure 3B). The ratio of M1:M2 cells within the fracture callus decreased with the progression of repair; however, this ratio was lower in aged animals compared to young animals, indicating a decreased injury-induced inflammatory macrophage response in aged animals.

In young mice, an initial drop in neutrophil population after fracture was followed by an increase to a level that was equal to or greater than what was seen in intact bone (Figure 3C). Conversely, in aged mice the number of neutrophils at the site did not change in response to fracture injury. B cell number was depleted in all groups after injury; this pattern was most pronounced in fracture calluses from aged mice (Figure 3D).

Fracture injury also generated notable changes in immune cell populations among PBMC’s in circulation. The number of CD3+ T cells in circulation decreased in response to fracture injury in young and old animals (Supplementary Figure S6A). Neither M1 macrophage nor M2 macrophage numbers changed significantly in the circulation, although M1 macrophage numbers trended towards an increase after injury (Supplementary Figure S6B). The number of neutrophils in circulation increased after fracture injury in aged animals of both sexes (Supplementary Figure S6C). B cells number declined in response to fracture injury throughout this inflammatory stage (Supplementary Figure S6D).

Importantly, no sex-dependent changes in immune cell populations were noted neither within the fracture callus (Supplementary Figure S7) nor within the circulation (Supplementary Figure S8) during fracture healing.

Collectively these data indicate an age-dependent change in cell recruitment and differentiation after fracture injury.

### Inflammatory cytokines are elevated in bone but not in circulation with advanced age

To determine if inflammatory cytokines are modulated with age, we used multiplex ELISA to test cytokines previously shown to be important in fracture healing. Midshaft tibial lysates and plasma samples from unfractured, young and aged, male and female mice were tested.

**Figure 4:**
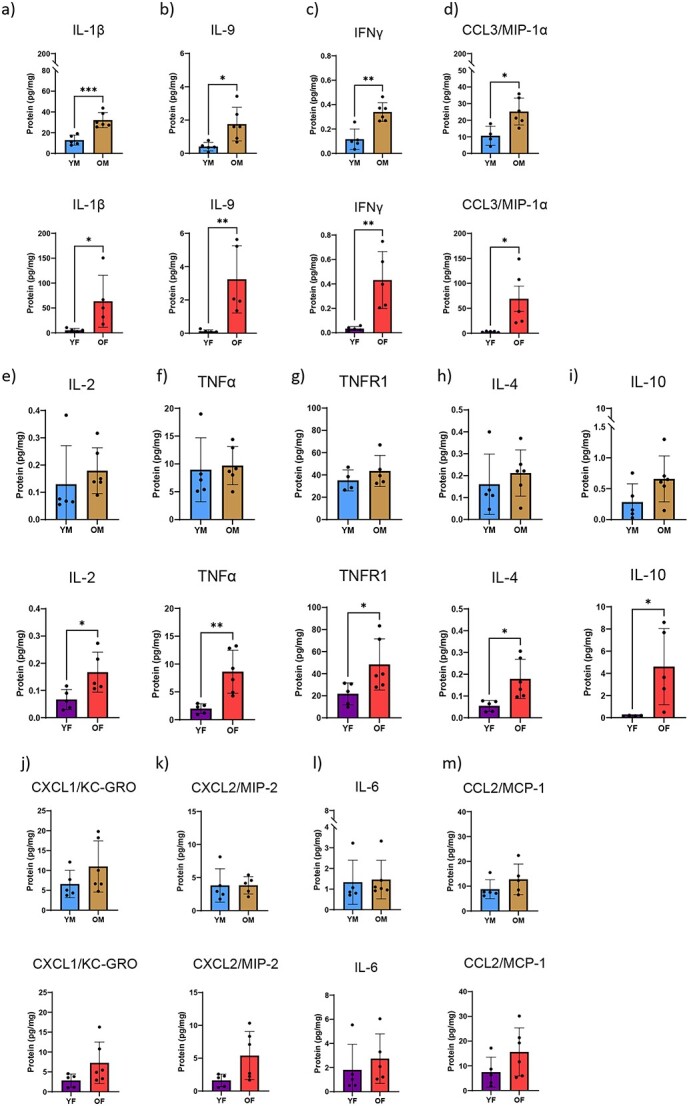
The cytokine profile within bone changes with age. Lysates of tibial diaphyses from uninjured mice were investigated for cytokine profile using multiplex ELISA. Calculated cytokine amounts were normalized to total amount of protein within the lysate. (A) IL-1β; (B) IL-9; (C) IFNγ; (D) CCL3/MIP-1α; (E) IL-2; (F) TNFα; (G) TNFR1; (H) IL-4; (I) IL-10; (J) CXCL1/KC-GRO; (K) CXCL2/MIP-2; (L) IL-6; and (M) CCL2/MCP-1 were determined from lysates of young and old, male and female mice. *N* = 5–6; two-tailed t-tests were conducted. ^*^P < 0.05, ^*^^*^P < 0.01, ^*^^*^^*^P < 0.001 (YM, young males; OM, old males; YF, young females; OF, old females).

Among bone lysates, concentrations of IL-1β, IL-9, IFNγ, and CCL3/MIP-1α were all found to increase with age in both male and female mice (Figure 4A–D). In addition, IL-2, TNFα, TNFR1, IL-4, and IL-10 levels increased with age but these increases were only significant in female mice (Figure 4E–I). There was a slight increase in levels of CXCL1/KC-GRO, CXCL2/MIP-2, IL-6, and CCL2/MCP-1 however these were not statistically significant (Figure 4J–M). Among plasma samples, IL-9 and IL-4 were undetectable in all four animal groups while all other cytokines were measurable. With the exception of TNFα levels, which increased with age in both males and females, and IL-10 levels, which increased in aged females, there were no significant age-associated differences (Supplementary Figure S9).

Sex-dependent analysis revealed disparate production of a group of cytokines in homeostatic bone. IL-1β, IL-9, CCL3/MIP-1α, and TNFα were found in lower abundance in young females compared to young males (Supplementary Figure S10). Importantly, all of these differences were lost with age. The only biomarker displaying differentiational sex-dependent expression with age was IL-10. IL-10 expression was higher in the bones of aged females than aged males (Supplementary Figure S10I). Within circulation, IL-10 was the sole cytokine differentially expressed in a sex-dependent manner. The circulation of aged females contained higher amounts of IL-10 relative to the circulation of aged males (Supplementary Figure S11).

**Figure 5:**
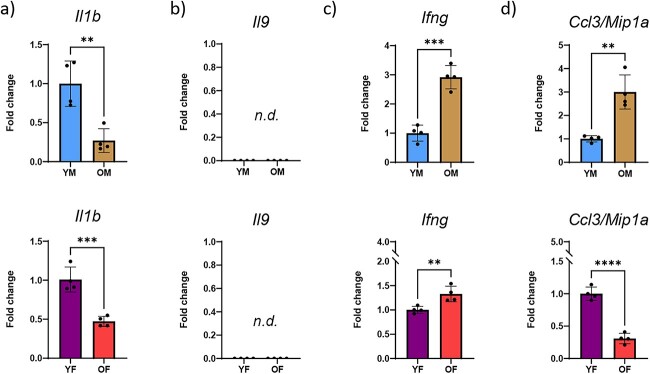
Transcript levels of age-accumulated cytokines fluctuate within bone tissue. Lysates of tibial diaphyses from uninjured mice were investigated for transcript levels of the cytokines which were found to be elevated with age using RT-PCR. (A) *Infg*, (B) *Cccl3/Mipa*, (C) *Il9*, and (D) *Il1b* were investigated from lysates of young and old, male and female mice. *N* = 5-6; two-tailed t-tests were conducted. ^*^^*^P < 0.01, ^*^^*^^*^P < 0.001. (YM, young males; OM, old males; YF, young females; OF, old females; *n.d.*, not detected).

These data confirm an age-associated increase of cytokines in bone tissue. It is not clear however whether these cytokines were produced locally within bone or whether they were produced elsewhere, secreted for systemic circulation, and then accumulated within bone over time. To answer this question, we measured transcript expression of the cytokines that were differentially present in bone tissue. Protein expression of IL-1β, IL-9, IFNγ, and CCL3/MIP-1α was significantly increased with age in bone tissue; however, transcript expression was less straight-forward. *Il1b* transcript decreased with age in both male and female bone tissue while *Il9* transcript levels were not detected (*n.d.*) in bone tissue (despite our use of 3 different primer pairs) (Figure 5A and B). *Ifng* and *Ccl3/Mip1a* transcript expression were increased 3-fold with age in male mice though this pattern was less robust for *Ifng* and reversed for *Ccl3/Mip1a* in female mice (Figure 5C and D). Protein levels of CXCL1/KC-GRO, CXCL2/MIP-2, IL-6, and CCL2/MCP-1 in bone tissue increased with age but were not significantly different. Interestingly, of these, *Cxcl1/Kcgro* and *Ccl2/Mip2* levels were significantly decreased with age in bone tissue from male and female mice (Supplementary Figure S12). Conversely, bone tissue transcript levels of *Il6* and *Ccl2/Mcp1* increased with age in male mice but decreased in female mice (Supplementary Figure S12).

Sex-dependent analysis of transcripts revealed that *Il1b* and *Ifng* were expressed higher in young males than in young females; however, the *Il1b* expression pattern reversed with age while *Ifng* differences were lost with age (Supplementary Figure S13). Conversely, *Ccl3/Mip1a*, *Il6*, and *Ccl2/Mip2* were expressed higher in the bones of young females than of young males. The *Ccl3/Mip1a* expression pattern reversed with age while the *Il6* and *Ccl2/Mip2* trends were lost with age.

These findings demonstrate that there is an age-associated accumulation of inflammatory cytokines within bone tissue and confirm that there are sex-related differences in young and aged animals; furthermore, the tissues of origin of these cytokines appear to vary.

### Cytokines categorize into either age-accumulated cytokines, female-specific age-accumulated cytokines, or fracture response cytokines

**Figure 6:**
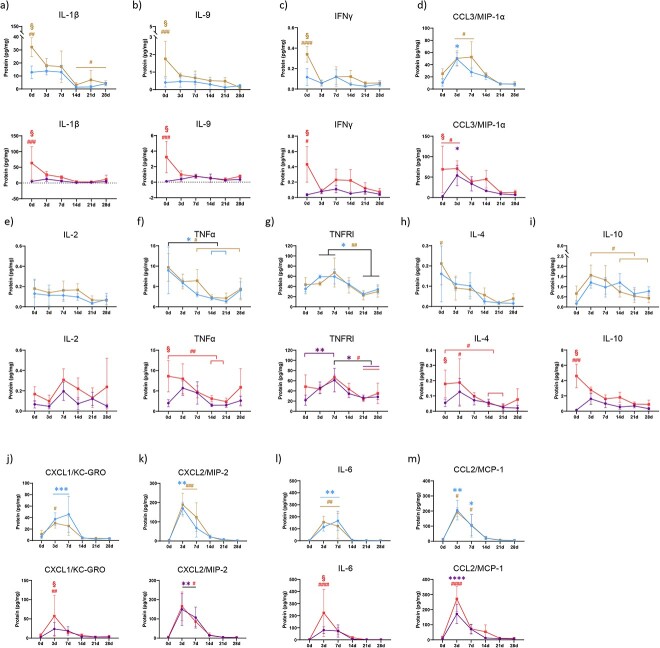
Age-dependent changes are present in cytokine response to fracture injury. Mice underwent tibial fracture surgery and fracture calluses were harvested 0dpf (prior to fracture), 3dpf, and 7dpf. Calluses were homogenized and investigated for cytokine profile using multiplex ELISA. Calculated cytokine amounts were normalized to total amount of protein within the lysate. (A) IL-1β; (B) IL-9; (C) IFNγ; (D) CCL3/MIP-1α; (E) IL-2; (F) TNFα; (G) TNFR1; (H) IL-4; (I) IL-10; (J) CXCL1/KC-GRO; (K) CXCL2/MIP-2; (L) IL-6; and (M) CCL2/MCP were determined from lysates of young and old, male and female mice. *N* = 5–6; data analyzed by two-way ANOVA followed by Tukey’s test when significant; ^*^P < 0.05, ^*^^*^P < 0.01, ^*^^*^^*^P < 0.001, ^*^^*^^*^^*^P < 0.0001 (YM, young males; OM, old males; YF, young females; OF, old females).

Fracture callus lysates from young and aged mice were investigated for cytokine levels using multiplexed ELISA. As we saw in unfractured bone, IL-1β, IL-9, IFNγ, and CCL3/MIP-1α levels increased in intact bone with age in both male and female mice. Immediately after fracture injury, these levels dropped and remained low even at 28dpf within the newly formed fracture callus (Figure 6A–D).

In intact, unfractured tibiae, all cytokine levels (IL-2, TNFα, TNFR1, IL-4, and IL-10) increased with age but this increase was only significant in bone tissue from female mice. Of these, IL-2, TNFα, IL-4, and IL-10 levels decreased after injury and did not return to the levels observed pre-fracture. TNFR1 level increased immediately after fracture injury in all groups and returned to baseline by 21dpf (Figure 6E–I). CXCL1/KC-GRO, CXCL2/MIP-2, IL-6, and CCL2/MCP-1 level all increased with age in uninjured bone but were not significant. All of these cytokine levels increased within injured/healing tissue immediately after bone injury, reached their maxima at 3 and 7dpf, and decreased thereafter (Figure 6J–M).

To account for the changes in cytokines within the fracture callus, transcript levels of cytokines were assessed. While IL-1β decreased in response to injury, *Il1b* transcript expression significantly rose at 3dpf in aged males and females, and at 7dpf in young males and females while *Il9* transcript was still undetectable at all time points (Supplementary Figure S14A and B). Transcript levels of *Ifng, Ccl3/Mip1a,* and *Il6* were highly variable across fracture-repair timeline and showed no distinct pattern of expression across either age or sex (Supplementary Figure S14C, D, G). Consistent with cytokine expression, transcript levels of *Cxcl1/Kcgro*, *Cxcl2/Mip2, Ccl2/Mcp1* in aged mice significantly increased at 3 or 7dpf and returned to baseline levels in both males and females (Supplementary Figure S14E, F, H).

With the exception of *Cxcl2/Mip2*, which displayed a delayed expression in aged females relative to aged males, neither cytokine abundance (Supplementary Figure S15) nor cytokine transcript levels (Supplementary Figure S16) within the fracture callus significantly changed in a sex-dependent manner during fracture healing.

Collectively, these data indicate that while plasma cytokine profiles change minimally, local cytokines can be classified into 3 different groups: (1) age-accumulated cytokines, (2) female-specific age-accumulated cytokines, and (3) fracture response cytokines. Our findings are summarized in Table 1.

**Table 1:** Cytokine summary of findings.

**(A) Age-accumulated cytokines**						
Cytokine/ Chemokine	Principle source(s)	Inflammatory profile	Protein – Homeostatic Bone	Protein – Fx Response	Transcript – Homeostatic Bone	Transcript – Fx Response
IL-1β	M1 macrophages	Pro-inflammatory	↑ in aged	“Depleted”	↓ in aged	“Respond & return”
IL-9	Th9, Tc9, Th2 T cells	Pro-inflammatory	↑ in aged	“Depleted”	Not detectable	Not detectable
IFNγ	T cells	Pro-inflammatory	↑ in aged	“Depleted”	↑ in aged	Highly variable
CCL3/MIP-1α	M1 macrophages	Pro-inflammatory	↑ in aged	“Respond & Return”	↑ in OM, ↓ in OF	“R&R” in YM; “Depleted” in YF, OM, & OF
**(B) Female-specific age-accumulated cytokines**						
Cytokine/ Chemokine	Principle source(s)	Inflammatory profile	Protein Basal levels	Protein – Fx Response	Transcript – Homeostatic Bone	Transcript – Fx Response
IL-2	Th1, CD8+ T cells	Pro-inflammatory	↑ in aged, F only	No response	Not measured	Not measured
TNFα	Macrophages	Pro-inflammatory	↑ in aged, F only	“Depleted”	Not measured	Not measured
TNFRI	All cells	Pro-inflammatory	↑ in aged, F only	“Respond & return”	Not measured	Not measured
IL-4	CD4+, Th2 T cells	Primarily Anti-inflammatory	↑ in aged, F only	“Depleted”	Not measured	Not measured
IL-10	CD4+ T cells, B cells, M2 macrophages	Primarily Anti-inflammatory	↑ in aged, F only	“Depleted”	Not measured	Not measured
**(C) Fracture-response cytokines**						
Cytokine/ Chemokine	Principle source(s)	Inflammatory profile	Protein Basal levels	Protein – Fx response	Transcript – Homeostatic Bone	Transcript – Fx response
CXCL1/KC-GRO	M2 macrophages	Pro-inflammatory	No age/sex difference	“Respond & return”	↓ in aged	“Respond & return”
CXCL2/MIP-2	M2 macrophages	Pro-inflammatory	No age/sex difference	“Respond & return”	↓ in aged	“Respond & return”
IL-6	M1 macrophages, CD4+ T cells	Pro-inflammatory	No age/sex difference	“Respond & return”	↑ in OM, ↓ in OF	Highly variable
CCL2/MCP-1	M1 macrophages	Pro-inflammatory	No age/sex difference	“Respond & return”	↑ in OM, ↓ in OF	“Respond & return”

### Cytokines within plasma changed minimally in response to age and fracture injury

As with fracture calluses, plasma was collected to assess the levels of inflammatory cytokines within the circulation during fracture healing. Circulating cytokines changed minimally in response to fracture healing. IL-1β was the only cytokine in the plasma that increased over basal levels, which only occurred in young males at 7dpf (Supplementary Figure S17A). CCL3/MIP-1α, TNFα, IL-10, and IL-6 were significantly depleted during fracture healing, albeit only in aged females (Supplementary Figure S17D, F, I, L). All other cytokines in the circulation had no changes over time (Supplementary Figure S17).

Sex-dependent analysis revealed no differences in cytokine abundance within circulation after fracture injury (Supplementary Figure S18).

## Discussion

Age is one of the most significant predictors of bone health and of fracture outcome – older adults have weaker bones and higher rates of complication after bone injury such as delayed healing and non-union. Age-associated chronic inflammation has been postulated to be a significant contributor to these shortcomings. Here, we determined the effect of age and sex on immune cell populations and cytokines in homeostatic bone, fracture calluses, and in circulation using mouse models.

Advanced age altered the inflammatory cell profile in homeostatic bone: CD4+ helper T cells and CD8+ cytotoxic T cells were elevated in male mice. Conversely, the number of M1 macrophages and the number of M2 macrophages decreased in male mice. Importantly, no such age-dependent differences were identified in female mice. These age-dependent differences were lost upon fracture: CD4+ helper T cell numbers and CD8+ cytotoxic T cell numbers were similar between young and old fracture calluses 3 and 7dpf. Likewise, M1 macrophage and M2 macrophage numbers were not different between young and aged mice, with the exception of M2 macrophages 7dpf, when M2 macrophage number was significantly higher in fracture calluses from aged females compared to those from young females.

In co-culture models, it has been shown that CD4+ T cells induced expression of osteogenic markers and promoted mineralization of human BMSCs, whereas co-culturing with CD8+ cells inhibited this outcome.^32^ Furthermore, Reinke et al. have reported that terminally differentiated CD8+ T cells negatively affected bone regeneration in patients^33^ and that the ratio of CD4+ T cells to CD8+ T cells is an important factor in fracture healing and a potential node of intervention to improve bone regeneration in patients.^17^^,^^19^^,^^34^^,^^35^ In our study, CD8+ cytotoxic T cell number decreased dramatically in all groups after fracture and we observed lower CD4:CD8 ratios in fracture calluses from aged mice than in those from young mice. Here, we considered CD4+ T cells and CD8+ T cells to comprise the two major classes of T cells. While T cell populations can be further delineated, in this study we investigated the global inflammatory landscape as it pertains to age- and sex-dependent changes related to fracture healing and have reserved further nuanced T cell delineation for future studies.

Immediately after fracture injury, M1 macrophage numbers decreased in young male mice but remained unchanged in aged male mice while M2 macrophages increased in a similar rate in fracture calluses from both young and aged mice. These findings point to the importance of an age-dependent function of M1 macrophages that is often overlooked in the literature. Indeed, in our previous work, Maresin 1 treatment of aged mice decreased M1:M2 macrophage ratio within the fracture callus similar to the pattern seen here in young mice, leading to improved bone fracture healing.^24^ Based on work by us and others showing that M2 macrophages play an important role in bone homeostasis and fracture healing, it would seem that a larger M2 population would be expected in the fracture calluses of young animals^20^^,^^36–38^; however, an age-dependent change in M2, anti-inflammatory macrophage population was not observed. Our study here indicates that it is the shift in the M1:M2 ratio that promotes healing. Macrophage polarization exists along a continuum with M1 and M2 as the simplified, most extreme forms of differentiation.^39^ Thus, it is possible that there are age-dependent changes in subtypes of macrophage populations that fall outside the scope of this study. Furthermore, we have previously demonstrated that the secretome of macrophages changes with age to subsequently affect bone and muscle regeneration.^20^^,^^21^^,^^40^ Thus, more complex investigation of inflammatory cell subtypes is warranted, utilizing CyTOF and similar technologies to further delineate changes in age- and sex-dependent inflammatory cell response.

Interestingly, neutrophil and B cell numbers within the fracture calluses did not change immediately after fracture injury in any groups. However, neutrophils increased significantly at 7dpf in young mice while B cells decreased significantly 7dpf in aged mice. This coincides with our previous findings in which we identified an immediate increase in neutrophil number and associated extracellular vesicles to be critical in parabiosis-based rejuvenation of fracture healing.^25^ In both studies, neutrophils serve as the largest immune cell population post-injury and are in higher abundance in the callus than in peripheral blood. Importantly, young males have more circulating neutrophils than do aged males, indicating a potential role for neutrophils in age-dependent shortcomings in fracture healing. Work by others has found that a loss of B cells dysregulates matrix production in homeostatic bone.^17^ Indeed, here we demonstrate that B cell counts are significantly decreased within fracture calluses from aged mice and are associated with diminished fracture healing.

Cytokines are a key group of small signaling proteins secreted during and critical for the homeostasis and response of inflammatory cells. From our analysis of intact and healing tibiae, bone cytokines can be placed into three groups: (1) age-accumulated cytokines, (2) female-specific age-accumulated cytokines, and (3) fracture response cytokines.

### Age-accumulated cytokines

(IL-1β, IL-9, IFNγ, and CCL3/MIP-1α) are cytokines that are found in higher concentrations in bone tissue with age. For the most part, cytokine levels decreased significantly after fracture injury and did not return to elevated, basal levels in newly formed bone tissue.

We found that IL-1β followed this pattern although we did note that IL-1β levels increased after fracture in young mice but this was not significant. Indeed, others have reported a fracture-induced increase in IL-1β within the fracture callus.^41^ We determined that despite an increased level of bone IL-1β with age, *Il1b* transcript levels decreased in bone tissue with age. Interestingly, *Il1b* transcript levels increased within the fracture callus immediately after injury in all groups and subsided during healing. IL-1β has been shown to prevent progenitor cell differentiation to osteoblasts; however, neither a loss of IL-1β receptor expression nor treatment with IL-1β result in a significant fracture healing phenotype.^42^^,^^43^

IL-9 has been reported to be elevated in the plasma of rheumatoid arthritis patients and is associated with increased osteoclastogenesis^44^^,^^45^ but its role in fracture healing has yet to be investigated. We detected IL-9 protein in intact bone and healing bone; interestingly, IL-9 protein was not detected in plasma nor was *Il9* transcript detected in intact or healing bone. While IL-9 protein accumulates within bone during aging, it appears that it is produced elsewhere and accumulates within bone tissue.

We found that levels of IFNγ protein and *Ifng* transcript increased within bone tissue with age and that IFNγ protein levels decreased after fracture injury and remained low while *Ifng* transcript levels were highly variable within the fracture callus of all groups. This is likely due to the fact that IFNγ is a pro-inflammatory cytokine that is primarily made by T cells and recruits and induces *Ifng* expression within a multitude of cell-types. IFNγ has been shown to increase with age and is able to decrease osteoclast activity and fusion^46^^,^^47^; however, inhibition of IFNγ delays bone fracture healing.^48^

Out of the 4 age-accumulated cytokines, the exception to the pattern of significant decrease secondary to fracture was the concentration of CCL3/MIP-1α, which increased after fracture injury and decreased over time in both sexes. Similar to the other age-accumulated cytokines, CCL3/MIP-1α did not return to elevated, basal levels in newly formed bone tissue. Others have observed a similar trend of CCL3/MIP-1α level increase in response to fracture injury in patient hematomas.^49^^,^^50^

### Female-specific age-accumulated cytokines

(IL-2, TNFα, TNFR1, Il-4, and IL-10) are cytokines that are found in higher concentrations in bone tissue with age, in only females. Cytokine levels decreased significantly after fracture injury and did not return to elevated, basal levels in newly formed bone tissue. IL-2, TNFα, and TNFR1 are pro-inflammatory cytokines while IL-4 and IL-10 are anti-inflammatory cytokines. The role of IL-2 has not been identified in fracture healing; however, TNFα, IL-4, and IL-10 play concentration-dependent roles in robust bone repair.^1^^,^^11^^,^^35^^,^^51^ In plasma, TNFα expression was elevated with age in both males and females while IL-10 expression was elevated in aged females only. Age-associated increase in plasma TNFα and IL-10 has been associated with decreased bone mineral density and increased rates of osteoporosis; elevated plasma TNFα is now an established biomarker of frailty in older adults.^52^ This sex-disparate pattern has been observed in prior studies in which female patients and mice mounted a heightened inflammatory response to infection and injury.^53^^,^^54^ Interestingly, we found that TNFR1 accumulated in the bone tissue of aged female mice; however, levels of this pro-inflammatory cytokine increased during early stages of fracture healing. Ochi et al. found that TNFR1 is essential for osteoclast differentiation as well as osteoblast survival.^55^

Sex-dependent differences in fracture healing are broadly noted in the literature. Indeed, we report both young and aged female mice develop smaller fracture calluses (TV, total volume) than their male counterparts. A possible cause of this discrepancy is the lower body weight of female mice leading to decreased mechanical stimulation. However, to better understand the effect of sex on inflammatory function during homeostasis and during fracture healing, we assessed immune cell population dynamics and cytokine protein and transcript abundance. During homeostasis, sex-dependent differences were identified in immune cell populations and cytokine levels; however, these differences were lost with age. Perhaps, most importantly, no sex-dependent differences in immune cell population nor in cytokine levels remained upon inducing fracture injury. Differential healing between males and females likely occurs independent of inflammatory response while inflammatory response plays a larger role in age-dependent deficits of fracture healing than in sex-dependent differences of fracture healing.

### Fracture-response cytokines

(CXCL1/KC-GRO, CXCL2/MIP-2, IL-6, CCL2/MCP-1; the age-accumulated CCL3/MIP-1α and female-specific age-accumulated TNFR1 can also be included here) were found at the same low level in bone tissue in young and aged mice until fracture injury was induced, upon which their protein and transcript levels increased locally. CXCL1/KC-GRO serves as a neutrophil chemoattractant and has been shown to increase at the site of injury soon after fracture.^56^^,^^57^ CXCL2/MIP2 seems to function in a similar fashion to CXCL1/KC-GRO. Indeed, as in the literature, we found expression peaked during early stages of bone fracture healing and subsided thereafter.^58^ Inhibition of IL-6 in mouse models decreases inflammation and decreases immune cell recruitment, subsequently delaying fracture healing.^59^ CCL2/MCP-1 is involved in the conversion of pro-inflammatory macrophages toward an anti-inflammatory phenotype and is required during early stages of bone healing for bone formation.^60^^,^^61^

Taken together, a picture starts to develop connecting age-dependent cellular response within the fracture callus to age-accumulated cytokines present within the fracture callus. Patterns emerge relating the changes in cellular populations and the cytokines those cells produce (Table 1). T cells produce both pro- and anti-inflammatory cytokines; IFNγ, and IL-9 are pro-inflammatory, all accumulated only in age, and these proteins are “*depleted*” during the course of healing. IL-4 and IL-10 are both primarily anti-inflammatory, produced in CD4+ helper T cells, basally increased in aged females only, and also depleted after injury. More broadly, this speaks directly to the notion of “inflammaging” – the accumulation of cytokines with advanced age.

Fracture callus macrophages as well as the inflammatory gene subset they produce show a distinctly different pattern over the course of healing. In unfractured bone, cytokines from M1 pro-inflammatory macrophages show a clear response: transcription of *Il6*, *Ccl2/Mcp1*, and *Ccl3/Mip1a* are all higher in aged males vs. young males but lower in aged females vs. young females. While the levels of transcript vary over the course of healing, all three cytokines have a “*respond and return*” pattern of protein expression – sharply increasing at 3 and 7dpf and then returning to basal levels by 14dpf. Pro-inflammatory chemokines originating from M2 anti-inflammatory macrophages show a slightly different pattern of expression: transcription of *Cxcl1/Kcgro* and *Cxcl2/Mip2* are basally higher in all young animals, although there are no differences in protein levels of the unbroken bone. Over time, both transcription and protein expression react to the fracture with a “*respond and return*,” increasing at 3 and 7dpf and returning to basal levels by 14dpf. Importantly, in our study 3dpf represents the earliest timepoint of analysis, post fracture. While this timepoint does represent the inflammatory phase in fracture healing, it is possible that earlier events could have been missed in our study. Furthermore, here we use mouse models of stabilized tibial fracture healing which may not correlate linearly with other models of bone regeneration.

Here we have simultaneously investigated age- and sex-related inflammatory differences in fracture healing. While we show little-to-no differences in the cell- or cytokine-based systemic response, there are many significant differences in inflammatory cell and cytokine levels within the fracture callus. We find that advanced age leads to a low-level chronic inflammatory state within bone tissue. Fracture injury elicits a local inflammatory response that is augmented in an age- and sex-dependent manner. Identifying these differences is the first step in developing cell- and/or cytokine-based therapies to overcome age-dependent shortcomings in bone fracture healing.

## Supplementary Material

SupplementalFigures_ziae023

## Data Availability

The data underlying this article are available in the article and in its online supplementary material.
