# Supplementary material for: The impact of age and sex on the inflammatory response during bone fracture healing
Source: JBMR Plus. 2024 Feb 22;8(5):ziae023. doi: 10.1093/jbmrpl/ziae023 (PMC10978063; doi:10.1093/jbmrpl/ziae023)
Supplement: SupplementalFigures_ziae023 [file supplementalfigures_ziae023.docx]

**Supplemental Figure 1 – Advanced age diminishes bone fracture healing.** To perform an age-dependent assessment of fracture healing, young and old mice underwent tibial fracture surgery and healing tibiae were harvested to assess fracture repair. 21-day fracture calluses were investigated using micro-CT analysis to determine a) total callus volume (TV), b) bone volume (BV), and c) relative bone volume (BV/TV). 28-day fracture calluses were assessed using mechanical testing to determine d) structural stiffness and e) force to refracture. For all n=12, t-test; bars shown are ± S.D. *p<0.05, **p<0.01, ***p<0.001, ****p<0.0001. (YM, young males, blue; OM, old males, brown; YF, young females, purple; OF, old females, red)

**Supplemental Figure 2 – Sex-dependent analysis of fracture healing.** To perform a sex-dependent assessment of fracture healing, male and female mice underwent tibial fracture surgery and healing tibiae were harvested to assess fracture repair. 21-day fracture calluses were investigated using micro-CT analysis to determine a) total callus volume (TV), b) bone volume (BV), and c) relative bone volume (BV/TV). 28-day fracture calluses were assessed using mechanical testing to determine d) structural stiffness and e) force to refracture. For all n=12, t-test; bars shown are ± S.D., *p<0.05, **p<0.01, ***p<0.001, ****p<0.0001. (YM, young males, blue; YF, young females, purple; OM, old males, brown; OF, old females, red)

**Supplementary Figure 3 – Sex-dependent analysis of inflammatory cells within bone.** Tibial diaphyses from uninjured mice were homogenized and investigated for inflammatory cell populations using flow cytometry and related to total number of cells present within tissue. a) Percentage of CD3+, CD4+, and CD8+ T cells in the intact tibial diaphyses. b) M1 and M2 macrophages were measured and ratio of M1:M2 was determined. c) Neutrophils and d) B cells were measured within intact tibial diaphyses. For all, n=3; two-tailed t-tests were conducted. *p<0.05, **p<0.01, ***p<0.001, ****p<0.0001. (YM, young males, blue; YF, young females, purple; OM, old males, brown; OF, old females, red)

**Supplementary Figure 4 – Age-dependent immunophenotyping of PBMCs.** Blood was collected from uninjured mice and investigated for inflammatory cell populations using flow cytometry and related to total number of cells present within the sample. a) Total T cell number was measured using CD3 as a pan T cell marker; CD4+ helper and CD8+ cytotoxic T cell numbers were assessed. b) Macrophages were identified as CD11b+, Ly6G-. Pro-inflammatory macrophages (M1; Ly6c^high^) and anti-inflammatory macrophages (M2; Ly6c^low^) were assessed. c) Neutrophils were quantified as CD11b+, Ly6G+ cells and d) B cells were identified as CD19+ cells. For all, n=3; two-tailed t-tests were conducted. *p<0.05, **p<0.01, ***p<0.001, ****p<0.0001. (YM, young males, blue; OM, old males, brown; YF, young females, purple; OF, old females, red)

**Supplementary Figure 5 – Sex-dependent Immunophenotyping of PBMCs.** Blood was collected from uninjured mice and investigated for inflammatory cell populations using flow cytometry and related to total number of cells present within the sample. a) Total T cell number was measured using CD3 as a pan T cell marker; CD4+ helper and CD8+ cytotoxic T cell numbers were assessed. b) Macrophages were identified as CD11b+, Ly6G-. Pro-inflammatory macrophages (M1; Ly6c^high^) and anti-inflammatory macrophages (M2; Ly6c^low^) were assessed. c) Neutrophils were quantified as CD11b+, Ly6G+ cells and d) B cells were identified as CD19+ cells. For all, n=3; two-tailed t-tests were conducted. *p<0.05, **p<0.01, ***p<0.001, ****p<0.0001. (YM, young males, blue; OM, old males, brown; YF, young females, purple; OF, old females, red)

**Supplementary Figure 6 – Age-dependent immunophenotyping PBMC’s during fracture healing.** Mice underwent tibial fracture surgery and blood was harvested 0dpf (prior to fracture), 3dpf, and 7dpf and investigated for inflammatory cell populations using flow cytometry and related to total number of cells present within the sample. a) Total T cell number was measured using CD3 as a pan T cell marker; CD4+ cytotoxic and CD8+ helper T cell numbers were assessed within the CD3+ T cell population. b) Pro-inflammatory macrophage (CD11b+, Ly6G-,Ly6c^high^) and anti-inflammatory macrophage (CD11b+, Ly6G-, Ly6c^low^) number was measured. c) CD11b+, Ly6G+ neutrophils and d) CD19+ B cells amounts were measured within fracture calluses. For all, n=3; data analyzed by two-way ANOVA followed by Tukey’s test when significant; *p<0.05, **p<0.01, ***p<0.001, ****p<0.0001. (YM, young males, blue; OM, old males, brown; YF, young females, purple; OF, old females, red)

**Supplementary Figure 7 – Sex-dependent analysis of inflammatory cell response to fracture injury.** Mice underwent tibial fracture surgery and fracture calluses were harvested 0dpf (prior to fracture), 3dpf, and 7dpf. Calluses were homogenized and investigated for inflammatory cell populations using flow cytometry and related to total number of cells present within the fracture callus. a) Total T cell number was measured using CD3 as a pan T cell marker; CD4+ cytotoxic and CD8+ helper T cell numbers were assessed within the CD3+ T cell population. b) Pro-inflammatory macrophage (CD11b+, Ly6G-, Ly6c^high^) and anti-inflammatory macrophage (CD11b+, Ly6G-, Ly6c^low^) number was measured and M1:M2 ratio was determined. c) CD11b+, Ly6G+ neutrophils and d) CD19+ B cells amounts were measured within fracture calluses. n=3; data analyzed by two-way ANOVA followed by Tukey’s test when significant; *p<0.05, **p<0.01, ***p<0.001, ****p<0.0001. (YM, young males, blue; OM, old males, brown; YF, young females, purple; OF, old females, red)

**Supplementary Figure 8 – Sex-dependent immunophenotyping PBMC’s during fracture healing.** Mice underwent tibial fracture surgery and blood was harvested 0dpf (prior to fracture), 3dpf, and 7dpf and investigated for inflammatory cell populations using flow cytometry and related to total number of cells present within the sample. a) Total T cell number was measured using CD3 as a pan T cell marker; CD4+ cytotoxic and CD8+ helper T cell numbers were assessed within the CD3+ T cell population. b) Pro-inflammatory macrophage (CD11b+, Ly6G-,Ly6c^high^) and anti-inflammatory macrophage (CD11b+, Ly6G-, Ly6c^low^) number was measured. c) CD11b+, Ly6G+ neutrophils and d) CD19+ B cells amounts were measured within fracture calluses. For all, n=3; data analyzed by two-way ANOVA followed by Tukey’s test when significant; *p<0.05, **p<0.01, ***p<0.001, ****p<0.0001. (YM, young males, blue; OM, old males, brown; YF, young females, purple; OF, old females, red)

**Supplementary Figure 9 – Age-dependent cytokine profile of circulation.** Plasma of uninjured mice was collected and investigated for cytokine profile using multiplex ELISA. Calculated cytokine amounts were normalized to total amount of protein within the lysate. n=5-6; two-tailed t-tests were conducted. *p<0.05, **p<0.01, ***p<0.001. (YM, young males, blue; OM, old males, brown; YF, young females, purple; OF, old females, red; *n.d,* not detected).

**Supplementary Figure 10 – Sex-dependent analysis of cytokine profile within bone.** Lysates of tibial diaphyses from uninjured mice were investigated for cytokine profile using multiplex ELISA. Calculated cytokine amounts were normalized to total amount of protein within the lysate. n=5-6; two-tailed t-tests were conducted. *p<0.05, **p<0.01, ***p<0.001. (YM, young males, blue; YF, young females, purple; OM, old males, brown; OF, old females, red)

*n.d.*

*n.d.*

**Supplementary Figure 11 – Sex-dependent cytokine profile of circulation.** Plasma of uninjured mice was collected and investigated for cytokine profile using multiplex ELISA. Calculated cytokine amounts were normalized to total amount of protein within the lysate. n=5-6; two-tailed t-tests were conducted. *p<0.05, **p<0.01, ***p<0.001. (YM, young males, blue; YF, young females, purple; OM, old males, brown; OF, old females, red; *n.d,* not detected)

**Supplementary Figure 12 – Age-dependent cytokine transcript production in bone.** Lysates of tibial diaphyses from uninjured mice were investigated for transcript levels of the cytokines using RT-PCR. n=5-6; two-tailed t-tests were conducted. **p<0.01, ***p<0.001. (YM, young males, blue; OM, old males, brown; YF, young females, purple; OF, old females, red)

*n.d.*

*n.d.*

**Supplementary Figure 13 – Sex-dependent cytokine transcript production in bone.** Lysates of tibial diaphyses from uninjured mice were investigated for transcript levels of the cytokines using RT-PCR. n=5-6; two-tailed t-tests were conducted. **p<0.01, ***p<0.001. (YM, young males, blue; OM, old males, brown; YF, young females, purple; OF, old females, red; *n.d.*, not detected)

*n.d.*

*n.d.*

**Supplementary Figure 14 – Age-dependent cytokine transcript profile within fracture calluses during repair.** Lysates of fractured tibiae were investigated for transcript levels of the cytokines. a) IL-1β; b) IL-9; c) IFNγ; d) CCL3/MIP-1α; e) IL-2; f) TNFα; g) TNFR1; h) IL-4; i) IL-10; j) CXCL1/KC-GRO; k) CXCL2/MIP-2; l) IL-6; and m) CCL2/MCP cytokines levels were determined. n=5-6; two-tailed t-tests were conducted. **p<0.01, ***p<0.001. (YM, young males, blue; OM, old males, brown; YF, young females, purple; OF, old females, red; *n.d,* not detected)

**Supplementary Figure 15 – Sex-dependent analysis of fracture-callus cytokine abundance during healing.** Mice underwent tibial fracture surgery and fracture calluses were harvested 0dpf (prior to fracture), 3dpf, and 7dpf. Calluses were homogenized and investigated for cytokine profile using multiplex ELISA. Calculated cytokine amounts were normalized to total amount of protein within the lysate. n=5-6; data analyzed by two-way ANOVA followed by Tukey’s test when significant; *p<0.05, **p<0.01, ***p<0.001, ****p<0.0001. (YM, young males, blue; YF, young females, purple; OM, old males, brown; OF, old females, red)

*n.d.*

*n.d.*

**Supplementary Figure 16 – Sex-dependent cytokine transcript profile within fracture calluses during repair.** Lysates of fractured tibiae were investigated for transcript levels of the cytokines. a) IL-1β; b) IL-9; c) IFNγ; d) CCL3/MIP-1α; e) IL-2; f) TNFα; g) TNFR1; h) IL-4; i) IL-10; j) CXCL1/KC-GRO; k) CXCL2/MIP-2; l) IL-6; and m) CCL2/MCP cytokines levels were determined. n=5-6; two-tailed t-tests were conducted. **p<0.01, ***p<0.001. (YM, young males, blue; YF, young females, purple; OM, old males, brown; OF, old females, red; *n.d,* not detected)

**Supplementary Figure 17 – Age-dependent cytokine profile of circulation during fracture healing.** Mice underwent tibial fracture surgery and plasma was investigated for cytokine profile using multiplex ELISA. Calculated cytokine amounts were normalized to total amount of protein within the lysate. a) IL-1β; b) IL-9; c) IFNγ; d) CCL3/MIP-1α; e) IL-2; f) TNFα; g) TNFR1; h) IL-4; i) IL-10; j) CXCL1/KC-GRO; k) CXCL2/MIP-2; l) IL-6; and m) CCL2/MCP were determined from plasma of young and old, male and female mice. n=5-6; data analyzed by two-way ANOVA followed by Tukey’s test when significant; *p<0.05, **p<0.01, ***p<0.001, ****p<0.0001. (YM, young males, blue; OM, old males, brown; YF, young females, purple; OF, old females, red; *n.d,* not detected).

*n.d.*

*n.d.*

**Supplementary Figure 18 – Sex-dependent cytokine profile of circulation during fracture healing.** Mice underwent tibial fracture surgery and plasma was investigated for cytokine profile using multiplex ELISA. Calculated cytokine amounts were normalized to total amount of protein within the lysate. a) IL-1β; b) IL-9; c) IFNγ; d) CCL3/MIP-1α; e) IL-2; f) TNFα; g) TNFR1; h) IL-4; i) IL-10; j) CXCL1/KC-GRO; k) CXCL2/MIP-2; l) IL-6; and m) CCL2/MCP were determined from plasma of young and old, male and female mice. n=5-6; data analyzed by two-way ANOVA followed by Tukey’s test when significant; *p<0.05, **p<0.01, ***p<0.001, ****p<0.0001. (YM, young males, blue; YF, young females, purple; OM, old males, brown; OF, old females, red; *n.d,* not detected).

| Gene | Direction | Sequence (5'→ 3') |
| --- | --- | --- |
| *Ifng* | Forward | GCTTTGCAGCTCTTCCTCATG |
|  | Reverse | TCTTCCACATCTATGCCACTTGA |
| *Il1b* | Forward | GCAACTGTTCCTGAACTCAACT |
|  | Reverse | ATCTTTTGGGGTCCGTCAACT |
| *Il6* | Forward | TAGTCCTTCCTACCCCAATTTCC |
|  | Reverse | TTGGTCCTTAGCCACTCCTTC |
| *Il9 (1)* | Forward | ATGTTGGTGACATACATCCTTGC |
|  | Reverse | TGACGGTGGATCATCCTTCAG |
| *Il9 (2)* | Forward | AGACACCATGGACCTTATTTAAAATCTGAAG |
|  | Reverse | GGACACGTTATGTTCTTTAGG |
| *Il9 (3)* | Forward | CTCTCCGTCCCAACTGATGAT |
|  | Reverse | TGACGGTGGATCATCCTTCAG |
| *Ccl2/Mcp1* | Forward | TTAAAAACCTGGATCGGAACCAA |
|  | Reverse | GCATTAGCTTCAGATTTACGGGT |
| *Ccl3/Mip1a* | Forward | TTCTCTGTACCATGACACTCTGC |
|  | Reverse | CGTGGAATCTTCCGGCTGTAG |
| *Cxcl1/Kcgro* | Forward | TCTCCGTTACTTGGGGACAC |
|  | Reverse | CCACACTCAAGAATGGTCGC |
| *Cxcl2/Mip2* | Forward | CCAACCACCAGGCTACAGG |
|  | Reverse | GCGTCACACTCAAGCTCTG |
| *Gapdh* | Forward | GGTGAAGGTCGGTGTGAACG |
|  | Reverse | CTCGCTCCTGGAAGATGGTG |

Supplementary Table 1 – Primer sequences for real time PCR.
